# Functional cycle of EEA1-positive early endosome: Direct evidence for pre-existing compartment of degradative pathway

**DOI:** 10.1371/journal.pone.0232532

**Published:** 2020-05-01

**Authors:** Rimma Kamentseva, Vera Kosheverova, Marianna Kharchenko, Maria Zlobina, Anna Salova, Tatiana Belyaeva, Nikolay Nikolsky, Elena Kornilova

**Affiliations:** 1 Institute of Cytology of the Russian Academy of Sciences, St. Petersburg, Russia; 2 St. Petersburg State University, St. Petersburg, Russia; University of Jyvaskyla, FINLAND

## Abstract

Early endosomes, regarded as the main sorting station on endocytic pathway, are characterized by high frequency of homotypic fusions mediated by tethering protein EEA1. Despite intensive investigations, biogenesis of endosomes, boundaries between early and late endosomes, and process of cargo transition though them remain obscure. Here, using EGF/EGFR endocytosis as a model and confocal microscopy of fixed and live cells, we provide evidence favoring EEA1-vesicles being pre-existed vesicular compartment, that maintains its resident proteins’ level and is sensitive to biosynthetic, but not endocytic pathway disturbance. EEA1-vesicles directly fuse with incoming EGF/EGFR-vesicles into hybrid endosomes with separated EEA1- and EGFR-domains, thus providing a platform for rapid achievement of an excess of surface-derived membrane that is used to form intraluminal vesicles (ILVs). Thus, multivesicular structures colocalized with EEA1 are still early endosomes. “EEA1-cycle” ends by exclusion of EGFR-containing domains with ILVs inside that turns into MVE and restoration of initial EEA1-vesicles population.

## Introduction

Vesicular traffic is one of the basic processes allowing the cell to maintain its homeostasis, respond to external stimuli, coordinate signaling cascades in time and space, *etc*. By definition, vesicular traffic is a process of multiple macromolecular cargoes transportation from one cell compartment to another by means of membranous transport vesicles [1,2]. Two main highways of protein and lipid flow realizes in all cell types: biosynthetic/exocytic and endocytic ones. Main compartments of the first pathway are Endoplasmic Reticulum (ER) and Golgi Apparatus (GA). Being highly dynamic, these compartments, nevertheless, maintain their identity (*i*.*e*. protein/lipid composition, easy recognizable typical morphology, and specific functions), communicate with each other by directed flows of transport vesicles, and are thus considered as “pre-existing”.

On the contrary, endocytosed cargoes are detected only in vesicular structures of different size from about 100 nm up to several μm, some of them with tubular extensions or with multivesicular appearance, along whole endocytic degradative pathway. These structures are commonly known as endosomes. Earlier it was also found that the properties of endosomal membranes are changed as they pass from PM to juxtanuclear region. Though the degradative endocytic pathway was described in detail by electron and fluorescent microscopy and other approaches during last decades, biogenesis of endosomes and boundaries between endosomal subcompartments, mechanisms of cargo transition remain obscure. This is reflected in very conventional, indefinite classification of endosomes as “early” (EE) and “late” (LE) ones depending mostly on time of a cargo appearance in peripheral or juxtanuclear endosomal structures. Also, there are some recognizable features attributed to EE or LE. First, typical indicator of EE is the high capacity of homotypic fusions [3–5]. Then, at later stage the cargo is found in typical structures known as multivesicular bodies, or endosomes (MVB, MVE), which are often regarded as late endosomes (LE) able to fuse with lysosomes.

However, the principles of endocytic pathway organization as a whole are still under debates ([6,7], Bakker et al., 2017). On the basis of many studies of numerous cargoes’ endocytosis made with different approaches two main hypotheses have been formulated to explain organization of endosomal compartment. First one, known as endosomal carrier vesicles (ECV) hypothesis, has declared existence of two stable endocytic compartments, EE and LE, communicating through transport vesicles exactly as it is for biosynthetic/exocytic pathway. According to ECV hypothesis, small transport vesicles pinched off of different portals at PM then enter pre-existing EE, a structure/structures with tubulovesicular appearance, the main sorting station on endocytic pathway. Here, cargoes targeted to recycling accumulate in tubular parts and those to be delivered for lysosomal degradation are concentrated in vesicular entities that finally are detached from EE and give rise to MVB, which in essence are transport vesicles (or endosomal carrier vesicles, ECV) on the way from EE to LE [3]. Small GTPase Rab5 was identified as the key regulator of EE functioning, that recruits other effectors such as phosphatidylinositol-3-kinase Vps34 and tether protein EEA1, recognizing the both Rab5 and Vps34 product PI3P [8,9]. In terms of ECV hypothesis Rab5 and Rab7 and their binding partners are resident proteins of pre-existing EE and LE, respectively [10,11]. Importantly, as far as EEA1 mediates homotypic fusions of EE, both Rab5 and EEA1 are often recognized as identical markers for EE by default.

According to opposing “maturation model” [4,12], several newly formed endocytic vesicles fuse with each other soon after detachment from PM and all subsequent events occur within this single structure. These events include homotypic fusions with similar endosomes, exclusion of recycling cargo through tubular extensions at early steps and formation of intraluminal vesicles (ILVs) later on, thus transforming unilamellar vesicle into multivesicular one. This process was called “maturation” and implied gradual transformation of each EE into multivesicular LE believed to be governed by gradual exchange of Rab5 for Rab7 [13]. Recent experimental data have proved progressive manner of Rab5 to Rab7 conversion [14]. However, in terms of maturation, each next stage of transformation needs specific set of regulatory proteins and their binding partners that should be recruited from cytosolic pools. Thus, endosome is a transient structure, arising at PM and disappearing after fusion with lysosome.

Advances of last years result in discovering of new participants involved in regulation of endocytosis and understanding of many aspects of endosomes’ functioning that led to some convergence of the two main hypothesis [15,16]. Therefore, Rab5-dependent EEA1-mediated fusions of endocytic vesicles as early event and cargo sorting are recognized by the both hypothesis, also, maturation is often considered as the process of intraluminal vesicles formation (ILVs), however other aspects of endosomal apparatus functioning are still under debates. Until now, the questions where and how endocytic vesicles with cargo enter and exit EE or turn into EE are discussed. Respectively, in the context of the two hypotheses, the boundary between the early and the late endosomes is interpreted differently. Multivesicular structures arising at later stages of degradative pathway are considered to be transport vesicles from pre-existing early to late endosomes in ECV-hypothesis, whereas in the hypothesis of maturation they play the role of late endosomes *per se*. Still, the exact consequence of events concerning conversion of early endosomes into the late ones is obscure.

EGF receptor endocytosis is popular model to study regulation of cargo traffic along degradative pathway. EGF binding to its receptor (EGFR) stimulates the receptor tyrosine kinase (TK) and one of the first consequences is recruitment onto membrane of the endosomal vesicle and activation of RIN1, Rab5-specific GEF, immediately upon internalization [17,18]. In fact, EGF/EGFR complex drives its own endocytosis by initiating certain molecular events, among which Rab5 activation is the key one. Activity of PI3-kinase, localized in Rab5-dependent manner to endosomal membrane, creates binding sites for FYVE-domain containing proteins, among which is early endosomal autoantigen EEA1, a tether protein that has FYVE- and Rab5-binding domains at C-terminus and Rab5-binding domain at N-terminus, thus, allowing to anchor Rab5-positive structures at the first step of fusion process. According to maturation hypothesis, such Rab5 effector as EEA1 should be recruited onto newly formed EGFR-containing endosomes from cytoplasm and at some maturation stage dissociates back, being substituted by Rab7, master of late endosomal events. However, in terms of ECV hypothesis, endocytic vesicles acquire Rab5 but then fuse with pre-existing early endosomes constantly bearing both resident proteins, Rab5 and EEA1.

In ours and many other studies of endocytosis in different cell lines the population of EEA1-positive vesicles was detected in control, not stimulated by any internalized ligand, cells [19–22]. This usually is explained by the fact that EEA1 may be involved in the endocytosis of unlabeled serum growth factors. However, we have noticed that even in serum-starved cells with depleted growth factors (and therefore minimalized receptor-mediated endocytosis) population of EEA1-positive vesicles is always detected. Thus we address the question of EEA1 vesicular population identity and find out that (i) the amount of membrane-associated EEA1 protein does not changes during all course of simultaneously stimulated EGF/EGFR endocytosis; (ii) disturbance of biosynthetic, but not endocytic pathway affects appearance and number of EEA1 structures. Next, we have demonstrated that newly formed EGF/EGFR endocytic vesicles directly fuse with pre-existing EEA1-vesicle forming hybrid endosomes with separated EEA1- and EGF/EGFR-positive domains (iii). We believe, that these fusions provide an extra membrane, thus, accelerating ILVs formation from EGF/EGFR-domains. Also, we show that finally such hybrid endosome was segregated for empty EEA1-vesicles that restore initial EEA1-population and for tightly packed with EGF/EGFR vesicles, which we believe are typical MVE (iv). The data obtained favor EEA1-endosomes to be a stable, pre-existing compartment with vesicular appearance, which function, besides sorting, is fast and effective fusions providing an extra membrane area for cargo-loaded vesicles allowing formation of ILVs from EGF/EGFR-positive domains soon after endocytosis without severing membrane bilayer. For our knowledge, we for the first time provide direct evidence that EEA-positive vesicles fully meet the definition of a pre-existing early endosomal compartment.

## Results

### EEA1-positive vesicles in serum-starved HeLa cells: Membrane-associated EEA1 level does not depend on growth conditions

**Fig 1:**
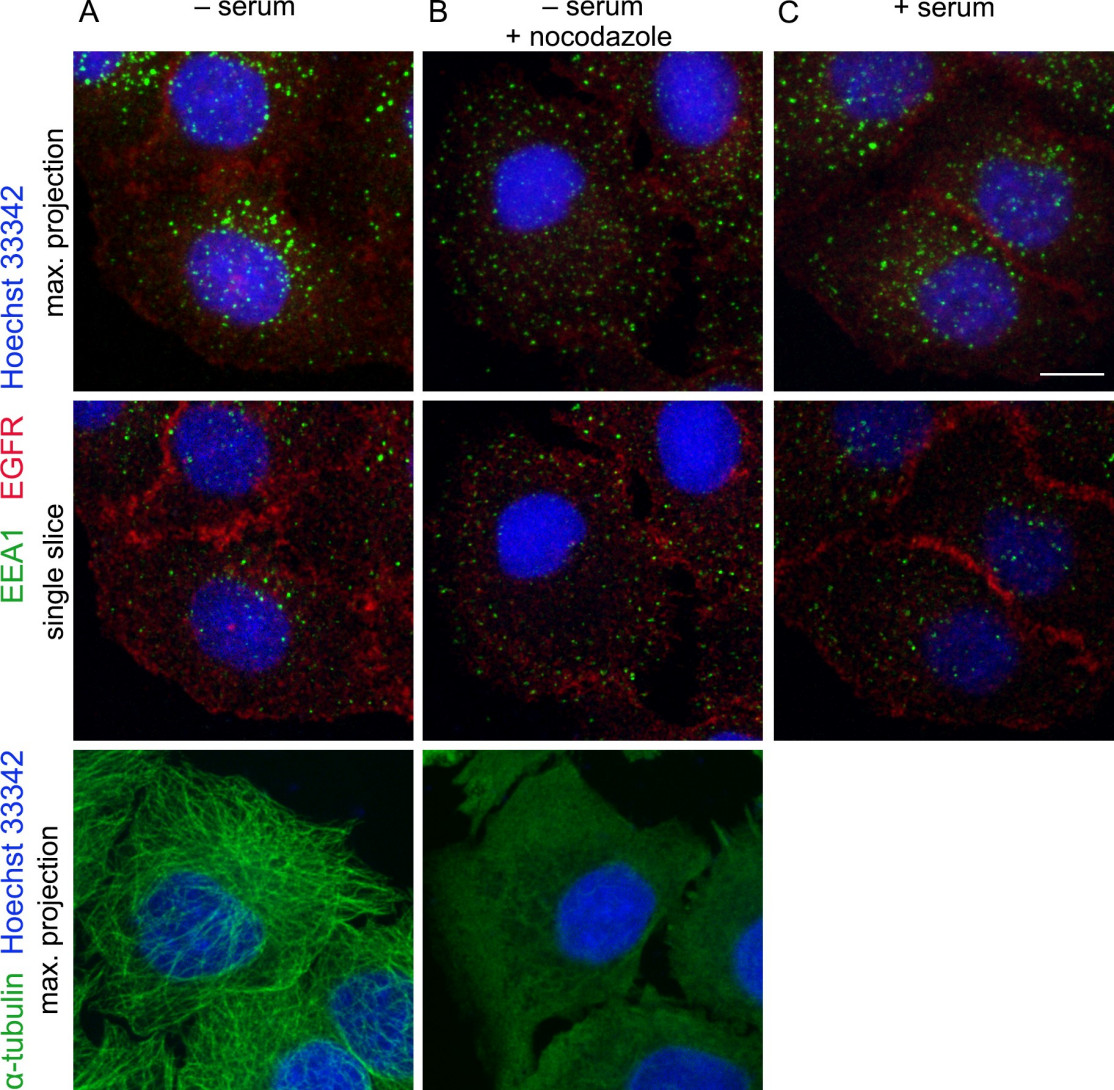
EEA1 is localized onto membrane vesicles independently on growth conditions. Hela cells were incubated in serum-free (A, B) or 10% FBS-containing (C) medium for 12h. (B) Cells were treated with 20μM nocodazole for 30 min before fixation. Then cells were stained using antibodies against EEA1 (green) and EGFR (red) (upper and middle row), or α-tubulin (lower row). Nuclei were visualized using Hoechst 33342 (blue). To visualize total population of EEA1-vesicles (upper row) and microtubules (lower row) the maximum intensity projections are presented. Single optical slices (middle row) are given to avoid possible artificial superposition of vesicles along Z axis. Scale bar—10 μm.

In case of EEA1, maturation hypothesis suggests that in the cells not stimulated for massive endocytosis EEA1 should be localized mostly in cytoplasm. However, incubation the cells in serum-depleted medium for 12 h to minimize receptor-mediated endocytosis of serum growth factors did not affect the appearance and number of bright EEA1-vesicles (Fig 1A). The same observation is found in numerous research articles [22–24]. As it is seen from MAX projection images these vesicles are tending to concentrate in juxtanuclear area of the cell, and this localization depends on microtubule network as after nocodazole treatment leading to MT depolymerization EEA1-vesicles become scattered throughout the cell, mostly peripherally (Fig 1B). EEA1-vesicles localization was mostly similar in serum-starved cells and those grown in full medium (Fig 1C). We have earlier demonstrated that they are Rab5-positive [20]. The same vesicles population can be seen in not-stimulated A549 cells (S2A Fig). Note, that independently of growth conditions cytoplasmic EEA1 staining is negligible. These does not exclude cytoplasmic localization of EEA1, but rather indicates that local concentration of membrane-associated EEA1 is higher than in cytoplasm. However, serum growth factors endocytosis is not a reason of EEA1 association with vesicular membranes.

We have reported earlier that 24 h serum starvation does not stimulate autophagy in HeLa cells and thus EEA1-positive structures found in serum-free cells are not autophagosomes that can also bear EEA1 [25]. Also, HeLa, as well as A549 cells, express EGF receptor at relatively high level, so ligand-independent internalization typical to EGFR overexpression may produce some endosomes. However, there were few receptor-positive vesicular structures in the cells not treated with EGF as well as receptor-positive/EGF-negative vesicles upon EGF endocytosis stimulation (Fig 4A, -EGF and S2A Fig, -EGF)

### EEA1 association with membranes is not sensitive to EGFR endocytosis stimulation

**Fig 2:**
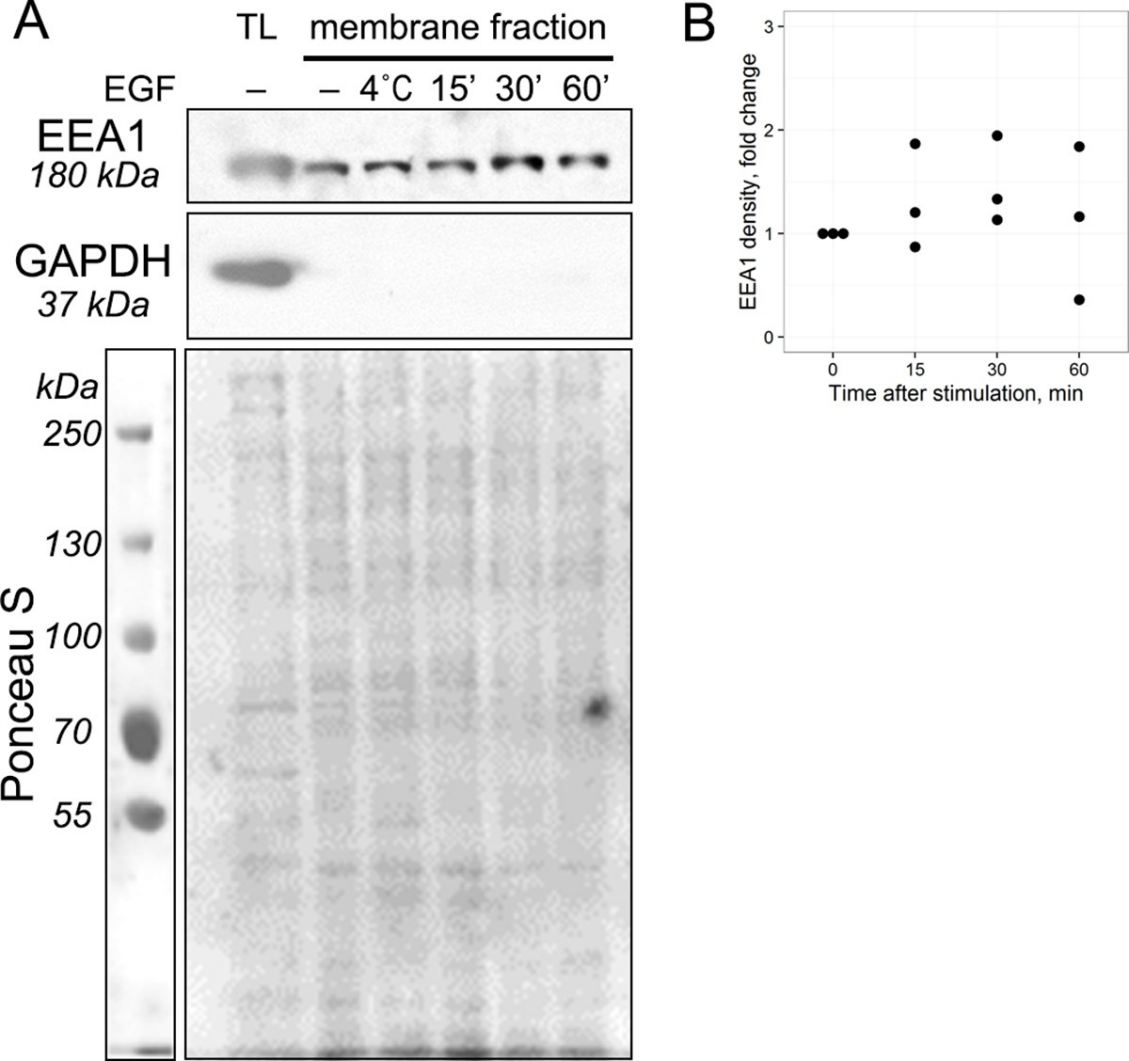
The amount of EEA1 detected in membrane fraction does not change after stimulation of EGF receptor endocytosis. EGF was allowed to bind to the membrane of HeLa cells at +4°C for 40 min and then EGFR endocytosis was stimulated by transfer to +37°C for the time indicated. (A) The membrane fraction was isolated using ultracentrifugation. Levels of EEA1 and GAPDH in cell membrane fractions and in total cell lysate were detected by immunoblotting. The total protein quantity was assessed by Ponceau S staining and used as a loading control. (B) The EEA1 density was quantified in 3 independent experiments, normalized by Ponceau S staining and the quantity relative to control calculated for each time point. Non-parametric one sample sign test with Bonferroni-Holm correction for multiple comparisons showed no significant difference from control in all time points (corrected p-values are 1, 0.75 and 1 for 15, 30 and 60 min, correspondingly).

Next, we questioned if massive endocytosis stimulation leads to alteration in the amount of EEA1 protein associated with the endosomal membranes. If EEA1 is recruited onto membranes of newly formed endocytic vesicles from cytosol, a significant increase in membrane-associated EEA1 level should be detected following synchronous stimulation of EGF endocytosis, because the number of newly formed endosomes and initial EEA1-vesicles are comparable (see Fig 4B and S2B Fig, 5 min). Also, membrane association of EEA1 is expected to decrease as early endosomes mature into late ones (30–60 min). To check this possibility, membrane fractions of the control (serum-starved) and EGF-stimulated HeLa cells were isolated by ultracentrifugation as described in Materials and Methods and its EEA1 content was analyzed by immunoblotting (Fig 2).

It is seen that there is no reliable difference in total membrane association of EEA1 before stimulation and during all 60 min of endocytosis. The absence of GAPDH in membrane fractions indicates that there is no cytosol contamination (Fig 2A).

However, in number of publications significant cytosolic pool of EEA1 was reported [26–28]. In our earlier work we have demonstrated by FRAP analysis that most of EEA1 proteins are able to cycle between cytosol and membranes with high rate, but the resulting balance is shifted toward membrane association state at normal conditions [29]. Practically constant level of EEA1 membrane staining indicates that most of binding sites are usually occupied. Anyway, these results indicate that massive EGFR endocytosis stimulation does not lead to any changes in the amount of EEA1 protein associated with the endosomal membranes and argued that cytosolic EEA1 pool is not significantly involved in mediating early endocytic events.

### EEA1-positive structures are sensitive to disturbance of biosynthetic but not endocytic pathways

As was mentioned above, in terms of the hypotheses under discussion, EEA1-positive vesicles may arise by two ways. In case of transient maturing single endosomes, endocytosis must play the key role in EEA1-vesicles formation. On the contrary, long-lived “pre-existing” structures have to be more sensitive to disturbance of biosynthetic pathway.

**Fig 3:**
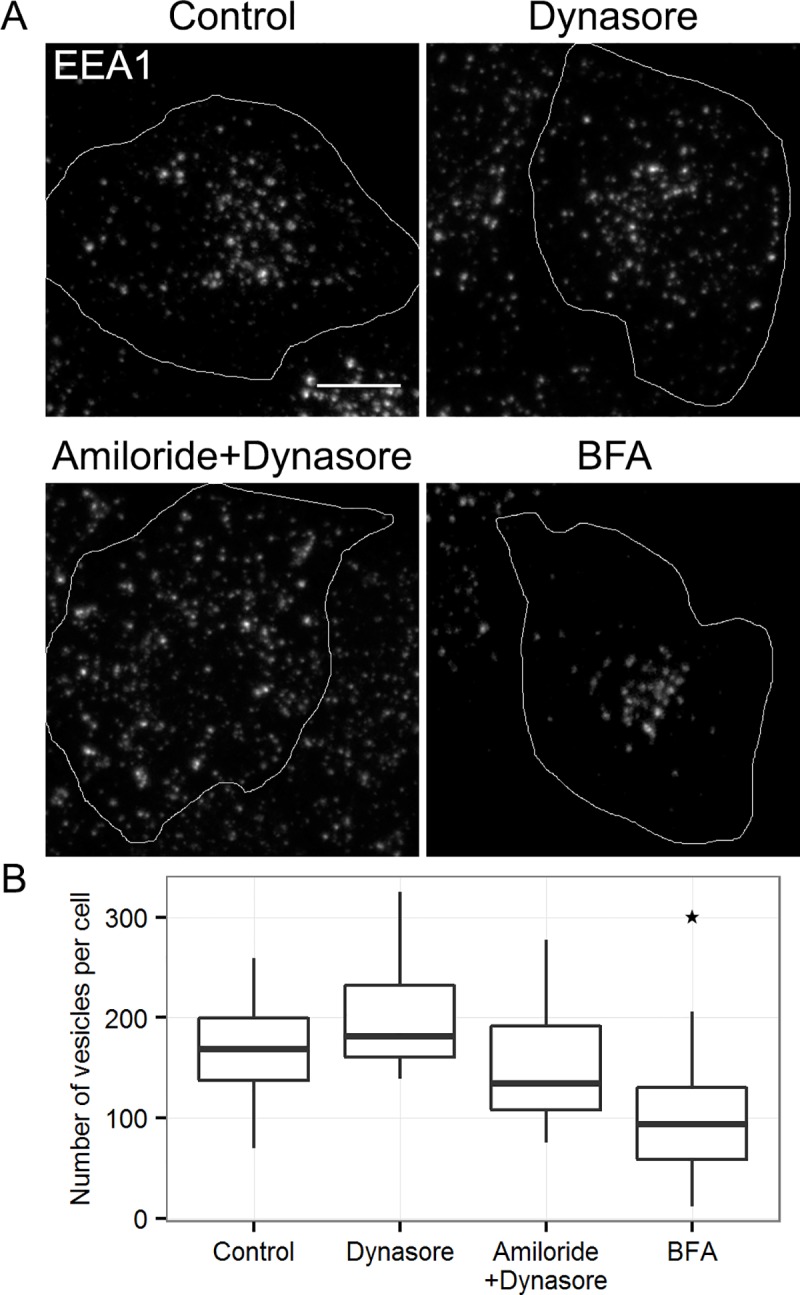
The effect of endocytosis inhibition and golgi disturbance on EEA1-positive vesicles. Hela cells were incubated for 6 hours with 10 μg/ml brefeldin A (BFA) to disrupt biosynthetic pathway, or 80 μM dynasore alone or in combination with 66 μM 5-(N, N-hexamethylene) amiloride to block endocytosis. Then cells were fixed and immunostained with anti-EEA1 antibodies. (A) The maximum intensity projections of typical cell are presented. Scale bar—10 μm. (B) The number of EEA1-vesicles was quantified in 15–20 cells per condition. The data are presented as boxplot that shows median, 25% and 75% quartiles, minimum and maximum value. The star indicates p<0.05.

In the next set of experiments we have analyzed whether inhibition of RME and/or non-selective endocytosis (by dynasore and amiloride) or disruption of Golgi functioning with brefeldin A (BFA) affects EEA1 population. Fig 3 represents the results of 6 h incubation of HeLa cells with dynasore or/and amiloride and BFA. We chose 6 h interval as a period enough both to degrade cargoes that have entered the cells before dynasore and amiloride treatment and to affect structures sensitive to Golgi severing but not enough for possible dramatic impact on the whole long-lived endocytic machinery. It is seen that dynasore in combination with amiloride treatment resulted in less compact localization of EEA1-positive vesicles (Fig 3A), possibly due to effect of dynasore on actin dynamics [30,31], but their number per cell was similar to that in control (Fig 3B). However, BFA treatment decreased the number of EEA1-positive vesicles. These data favor more biosynthetic rather than endocytic way of EEA1-vesicles biogenesis that is typical for intracellular compartments in general.

### Stimulation of EGFR endocytosis initiates the set of consecutive events mediated by hybrid EGFR-EEA1 organelles, or “EEA1-cycle”

**Fig 4:**
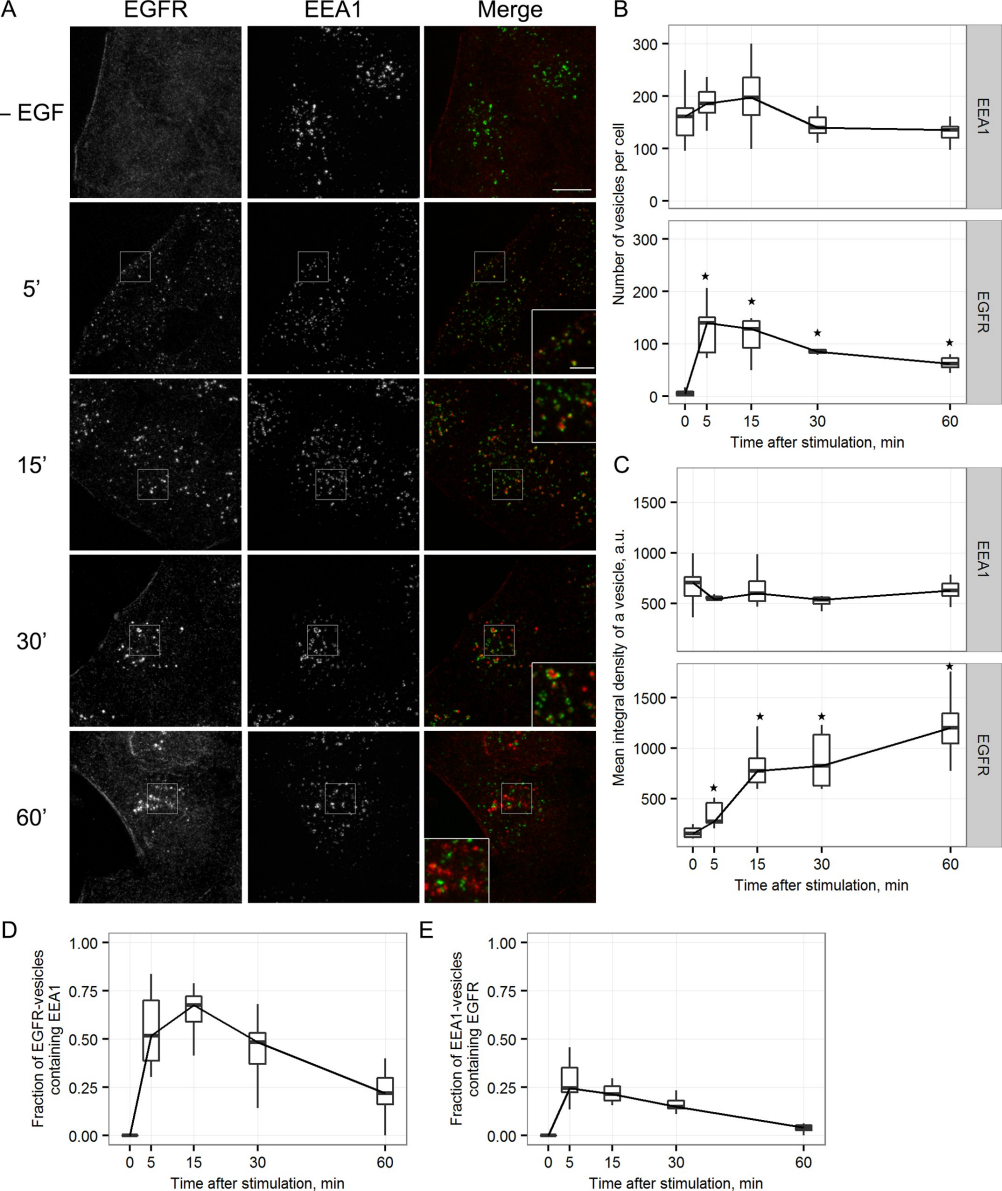
The dynamics of EEA1- and EGFR-positive vesicles colocalization in HeLa cells following the stimulation of EGF/EGFR-complexes endocytosis. Endocytosis in serum-deprived Hela cells was stimulated according to pulse-chase protocol by adding of EGF for 5 min followed by washout of unbound ligand and chase period at 37°C. Cells not treated with ligand (- EGF) and cells chased for the indicated period were fixed and immunostained using antibodies against EEA1 (green channel) and EGFR (red channel). (A) Maximum intensity projections of the typical cells are presented. Scale bar—10 μm (3 μm in the enlarged insets). The number (B), mean integral density of vesicles (C) and object-based colocalization of the cells from the same experiment (D, E) were quantified. For each time point 15–20 cells were taken into analysis. The data are presented as boxplot that shows median, 25% and 75% quartiles, minimum and maximum value. In B,C the star indicates significant difference from unstimulated cells (p<0.05).

In the case that EEA1-positive vesicular population is pre-existing compartment, EGF/EGFRs transport vesicles should enter it due to fusion and then exit as a result of fission process. We have analyzed in detail how do EGF/EGFR complexes transit through EEA1-positive early endosomes. In HeLa and A549 cells, EGFR is internalized after binding to its ligand EGF. To synchronize endocytic events we added EGF (40 ng/ml) to serum-starved cells by short 5 min-pulse at 37°C and after washing out unbound ligand continued incubation at 37°C for indicated time (Fig 4 and S2 Fig). At the end of each time interval the cells were fixed, stained with antibodies against EGFR and EEA1, and analyzed for localization of the proteins with confocal microscope. Typical images are presented in panel A of the both pictures. Also, the number of structures, their mean fluorescence intensities (panels B-C) as well as the object-based colocalization (defined as percentage of objects bearing first protein overlapping even partially with structures positive for another protein, normalized to the total number of the latter objects, as indicated in the legends to Fig 4 and S2 Fig, panels D and E) were estimated for at least 20–30 cells for every time point in each of at least 3 independent experiments.

Series of typical images of HeLa cells fixed at indicated time points after endocytosis stimulation (Fig 4A) shows that in absence of EGF EGFR was detected at the plasma membrane and not inside the cell, while EEA1 was associated with vesicles localized mostly in juxtanuclear region (Fig 4A,–EGF). Measurements show that there were, on the average, about 150 EEA1-vesicles per cell in the control (serum-starved) cells (Fig 4B). However, 5 min after endocytosis stimulation by pulse-chase protocol both EGFR and EEA1 were seen in very small dotted structures, the most of them localized just near the plasma membrane (Fig 4A, 5 min). Image analysis of the cells at this time point shows that fluctuations in the number and mean integral density of EEA1-vesicles during 60 min of experiment were within the statistical error in relation to control values for unstimulated cells, which correspond to the data on membrane-associated EEA1 fraction (Fig 2). However, in HeLa cells the number of EEA1-vesicles slightly increased by 5 and 15 min, while the mean integral vesicle density slightly decreased (Fig 4B and 4C). In expreiments with A549 cells presented in this paper (S2 Fig) this early fluctuations are even stronger remaining nonetheless statistically unreliable (S2 Fig, 5 min point). This tendency could be interpreted as the recruitment of a certain amount of EEA1 from the cytosol to the newly formed EGF/EGFR vesicles, which seems to support the maturation hypothesis. Note, however, that in this case it can be suggested that all incoming EGF/EGFR vesicles must acquire EEA1 to become able to fuse later. Nevertheless, in HeLa cells about 150 EGF/EGFR-containing vesicles were formed during first 5 min, but the number of EEA1vesicles grew only for 20 ones while colocalization degree of the two types of vesicles is about 50–80%. That means significant involvement of preexisting EEA1-vesicles. Once more, we would like to stress that these alterations were found to be statistically unreliable. Indeed, in many experiments on HeLa as well as on A549 cells we did not find any changes in the number of EEA1-vesicles as well as in their mean intensity after endocytosis stimulation. We rather suggest that such alterations might be due to reduction of EEA1-vesicles’ size as a result of fission events as it occurs under Nocodazole treatment, that produces more not so bright EEA1-vesicles at the periphery (Fig 1B). However, we did not exclude at all the possibility that some minor portion of cytosolic EEA1 become associated with newly formed EGF/EGFR-containing vesicles.

Colocalization analysis (Fig 4D and 4E) show that significant portion of EGF/EGFR- and EEA1-positive structures overlapped each other, but enlarged fragments of merged images (see corresponding insets in Fig 4) show that this is not “point-to-point” colocalization but rather there are EGFR- and EEA1-vesicles that maintain two-domain organization at 5 min. Different orientation of the two domains relatively each other clearly seen at the images proves that such two-domain structures are not a result of some systematic shifts between channels or chromatic shifts. It is well documented, that fusion process consists of several steps: tethering precedes reorganization of lipid bilayer. Usually tethering takes more time that the fusion itself and theoretically under synchronization conditions the stage of tethering can be detected. Unfortunately, fixed cell imaging cannot differentiate between tethering and final formation of a single vesicles with segregated domains.

**Fig 5:**
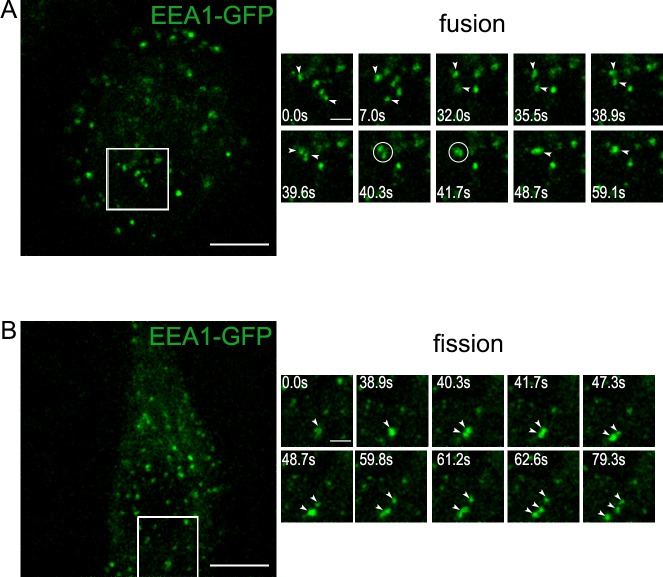
Live imaging of EEA1-vesicles population in serum-starved, non-stimulated Hela cells. The time-lapses present the fusion (A) and fission (B) events in serum-starved HeLa cells transiently expressing EEA1-GFP. The time after start of imaging is indicated at the frames. Scale bars– 10 μm (whole cell) or 3 μm (ROI time-lapse). Arrowheads indicate vesicles of interest, circle (A)–the moment of fusion. Full movies are presented as S1 and S2 Movies, respectively.

To clarify the details of these highly dynamic processes, and visualize all steps of EEA1- and EGF/EGFR-vesicles interactions in live HeLa cells we expressed plasmid encoding EEA1 fused to GFP (Addgene plasmid 42307, USA). In preliminary experiments we found that the expression of the plasmid does not affect the dynamics EEA1 and EGF/EGFR colocalization and delivery of EGF/EGFR to lysosomes (data not shown). We found that in control, serum-deprived cells with minimalized RME, most of EEA1-vesicles performed non-directional oscillatory movements in a limited area, but some of them were able to undergo fusions and fissions with each other, though the frequency of such events is relatively low (Fig 5A, S1 Movie, Fig 5B, S2 Movie). This indicates that EEA1-vesicles possess all machinery necessary for tethering and fusion.

**Fig 6:**
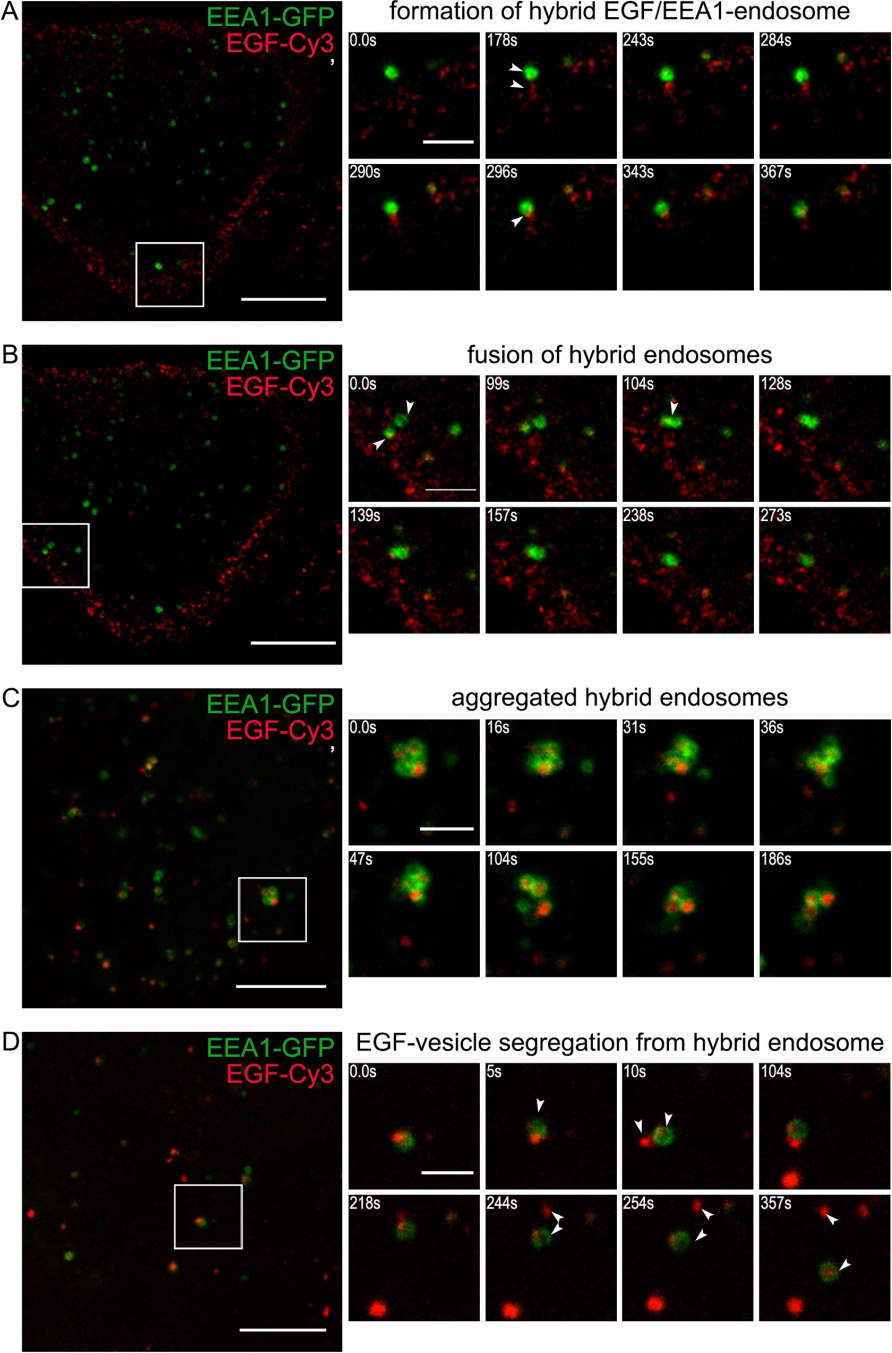
The EEA1-cycle in living HeLa cells expressing EEA1-GFP. EGFR endocytosis was stimulated by EGF-Cy3 (red channel) in HeLa cells transiently expressing EEA1-GFP (green channel) according to the pre-binding protocol (described in the Material and methods section) to synchronize endocytic events. Time-lapse imaging at +37°C was performed. The following stages of the cycle are presented: (A) formation of hybrid EGF/EEA1-endosome (5–10 min), (B) fusion of hybrid endosomes (5–15 min), (C) aggregated hybrid endosomes (15–40 min), (D) EGF-vesicle segregation from hybrid endosome (25–60 min). Scale bars– 10 μm (whole cell) or 3 μm (ROI time-lapse). Arrowheads indicate vesicles of interest. Full movies are presented as S3, S4, S6 and S8 Movies, respectively.

For the live imaging experiments endocytosis was stimulated by EGF bound to Cy3 or QD655 as indicated in Figure legends. EGF-Cy3 addition for 5 min at 37°C results in formation of EGF/EGFR complexes, and, as known, promotes their association with coated pits and coated vesicles. At this time point complex and highly dynamic pattern of EGF-Cy3 distribution can be seen mostly in close proximity of PM (Fig 6A, S3 Movie), with some of EGF-Cy3 localized in small structures with vesicular and tubular appearance. Video clearly demonstrates that during first 5–10 min such EGF-Cy3-containing vesicles contact and then become united with a nearby EEA1 vesicles giving rise to two-domain hybrid endosome, that is detected in our experiments on fixed cells at 5 min. So, we conclude that registered two-domain structures are not tethered EEA1- and EGF/EGFR-vesicles, but really fused hybrid endosomes bearing both EGF/EGFR and EEA1 in separated domains.

Formation of two-domain structure is the first step of EEA1-cycle. Live imaging also show that such two-domain endosomes are able to accept several vesicles (both EEA1- and EGF/EGFR-positive) for next several minutes thus turning into multi-domain enlarged hybrid vesicle. Next, neighboring two- or already multi-domain vesicles can fuse with each other further increasing the size of resulting endosome (Fig 6B, S4 Movie).

Importantly, we cannot completely exclude that initially some of cytosolic EEA1 becomes associated with these vesicles, but, in any case, its amount is so negligible that cannot be detected: we have not registered gradual accumulation of EEA1 onto the surface of EGF-containing vesicles. As was mentioned above, after several cycles of fusions between hybrid organelles the maximal colocalization between EEA1 and EGF/EGFR is achieved. According to estimation presented in Fig 4 and S2 Fig it is about 70%. Usually all these events take place during first 15–20 min of endocytosis, and, as can be seen from Fig 4 and 4C and S2 and S2C Fig, mean integral density of EGF/EGFR-domains significantly increases, indicating the process of cargo concentration.

By 15–30 min the appearance of the hybrid structures changed from two-domain to more complicated pattern (Fig 4A, 15–30 min) presented mostly by 3 types. It can be (a) a few two-domain vesicles, (b) single vesicles with multiple separated EGFR- and EEA1-positive domains (multi-domain vesicles) and (c) several apparently enlarged multi-domain structures of irregular shape (see corresponding images in Fig 4, and insets), with significant colocalization between EGFR and EEA1 signals. However live imaging clearly shows (Fig 6C) that such enlarged structures are not single vesicles, but rather dynamic aggregates of multi-domain hybrid endosomes. The parts of aggregates are bound by very weak and flexible bonds, some vesicles can enter this aggregates while some other left them. By 30 min such aggregates may also contain only EGF/EGFR- or EEA1-vesicles. Aggregation may indicate the achievement of a special stage at which membranes bearing EEA1 yet can tether each other, but are no longer able to fuse.

At 40–60 min irregular EGF/EGFR- and EEA1-positive structures are localized in juxtanuclear region (Fig 4A, 60 min, Fig 4D and 4E) It is important to note that, despite similar localization, the two proteins were associated mainly with different vesicular structures with minimal colocalization, while some of them seems to be in aggerated state. Decrease in the number of aggregated hybrid endosomes and appearance of non-colocalized of EFG/EGFR and EEA1 suggest active segregation of hybrid endosomes for only EGF/EGFR-containing and initial EEA1- positive vesicles. Thus, at 40–60 min of endocytosis “EEA1-cycle” is completed.

What is more important, our quantitave estimations (Fig 4B and 4C) show very slight alterations in number of EEA1 structures and in their fluorescence intensity during “EEA1 cycle” that also indicates that amount of EEA1 associated with membranes does not increased significantly. On the contrary, the number of EGF/EGFR-containing structures demonstrates initial fast growth during first 5–10 min followed by decrease while mean fluorescence intensity of vesicles grows all time of experiment, but most rapidly during first 15 min, when colocalization with EEA1 is maximal. This behavior fits permanent concentration process of EGFR-receptor complexes due to multiple EEA1-mediated fusions that correlates with multi-domain endosome formation. Behavior of EGF/EGFR-containing vesicles and EEA1-positive structures in HeLa is very similar to that in A549 with minor differences (compare Fig 4 and S2 Fig, also see [32]). To our experience that can be due not so to difference between cell types but rather reflect fluctuations in dynamics of endocytosis from one experiment to another.

However, small sizes of vesicular structures of interest did not allow investigating details of “EEA1-cycle” thoroughly. To clarify the point, we use the cell line of porcine aortic endothelium PAE A11 that constitutively expresses EGFR fused to GFP at cytoplasmic C-terminus of the receptor [33]. During long time of cultivation initial cell population becomes quite heterogenous in chimeric EGFR expression level. We note that the cells expressing EGFR-GFP at very high levels have two important features: first, fluorescent EGFR labels practically all membranes of organelles that are normally involved in the receptor synthesis/maturation/turnover, that are Golgi, exocytic vesicles, plasma membrane and endosomes of different nature, making them visible. Second, though in PAE A11 with relatively low EGFR-GFP expression EGFR undergoes normal endosomal processing [33], in receptor-overexpressing cells endosomal compartments were enlarged. The exact reason of this enlargement is not so important for our study, because early fusion processes proceed normally in PAE A11 cells and, as a result, small newly formed EGFR-positive vesicles form hybrid endosomal structures with EEA1 vesicles that are significantly enlarged in size.

**Fig 7:**
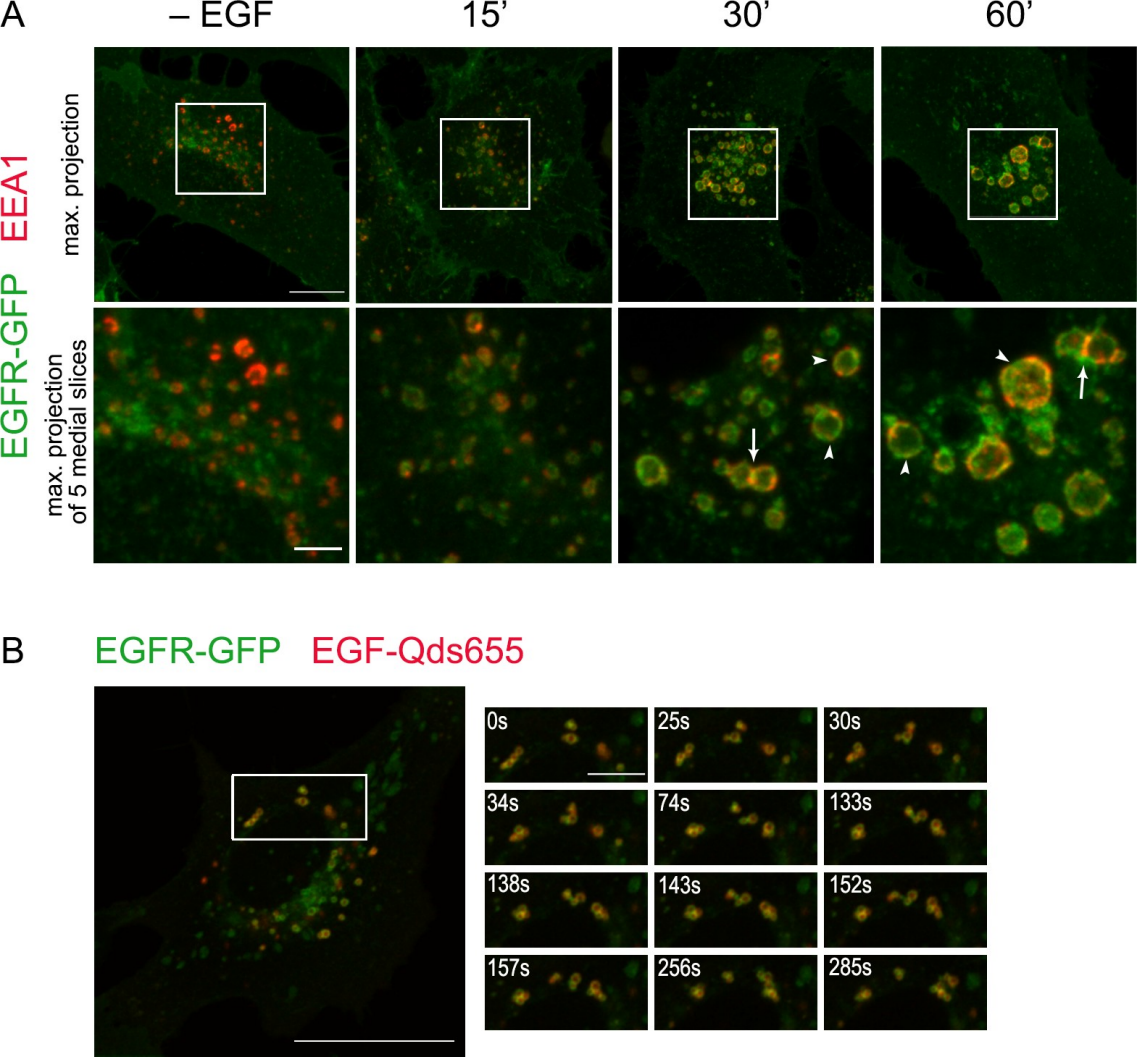
Localization of EEA1 protein on endosomes of PAE A11 cells expressing EGFR-GFP during endocytosis of EGFR. (A) EGFR endocytosis was stimulated in serum-deprived PAE A11 cells expressing EGFR-GFP (green channel) according to pulse-chase protocol by adding of EGF for 5 min followed by washout of unbound ligand and chase period at 37°C. Cells not treated with ligand (- EGF) and cells chased for the indicated period were fixed and immunostained using antibodies against EEA1 (red channel). Maximum intensity projection of the typical cells (upper row, scale bar—10 μm) and maximum intensity projection of 5 optical slices for the enlarged region (lower row, scale bar—3 μm) of typical cell are presented. Arrows indicates the sites of tethering between two vesicles, enriched with EEA1; arrowheads show the examples of vesicles with multiple EEA1-positive domains. (B) EGFR endocytosis was stimulated according to pulse-chase protocol by adding of EGF-QD655 for 5 min followed by washing out the unbound ligand and time-lapse imaging at +37°C started in 24 min after endocytosis stimulation. The chosen frames are shown and the time after start of imaging is indicated at the frames. Scale bars– 10 (whole cell) and 3 (ROI time-lapse) μm. Full movie is presented as S7 Movie.

Earlier we have shown that overexpressed EGFR is internalized constitutively to a rather high level [34]. However, in control, not EGF-treated cells, EEA1-positive vesicles bear very small or do not bear EGFR-GFP signal at all (Fig 7A,—EGF). To analyze EEA1 behavior in this cells, unlabeled EGF (50 ng/ml) was added according to pulse-chase protocol and in 15, 30 and 60 min after endocytosis stimulation the cells were fixed and stained with antibody against EEA1. Upper panel of Fig 7A presents projections of maximal intensity (Z-stacks) of PAE A11 cells and lower panel demonstrates enlarged insets with projections of maximal intensity for 5 optical sections that correspond to the size of large hybrid endosomes.

It is clearly seen that in large membrane profiles the EGFR-GFP-positive domains interspersed with the domains positive for EEA1. In an enlarged inset of endosomes about 3–5 such EEA1 inclusions can be seen supposing that a whole surface of such hybrid vesicle may be composed of membranes of several EEA1- and several EGF/EGR-vesicles united in larger domains (Fig 7A, 15 min). Importantly, there are several vesicles contacted with each other and EEA1 antibody intensively stains the sites of contact. It is also can be seen that at 30–60 min hybrid endosomes form tethered through their EEA1-domains clusters, that may correspond to irregular shaped structures found at this time in HeLa and A549. Presented video shows that the behavior of clustered hybrid endosomes is very similar to that in HeLa (S6 and S8 Movies).

As were mentioned above, by 60 min aggregates in HeLa cells mainly disintegrated and some hybrid endosomes as well as single vesicles that bearing only EEA1 and only EGF/EGFR can be seen. Thus, we speculate that segregation of hybrid endosomes back into separate vesicles occurs but it cannot be excluded that EGF/EGFR vesicles just lost gradually EEA1 due to its dissociation back to cytoplasm. However, when this process was monitored in live HeLa cells expressing EEA1-GFP it was clearly seen that EGF-Cy3-containing vesicles are excised from membrane of hybrid endosome (Fig 6D, S8 Movie). Several cycles of segregation result in complete removal of EGF-marked membranes. More often EGF/EGFR-vesicles rapidly move away from maternal structure suggesting motor-mediated translocation along microtubules. However, this fast linear run may be not so ultimate and the newly formed EGF/EGFR-containing vesicle can again get into contact with the just abandoned endosome, but back fusions seem to be forbidden. Thus, our data support the segregation process, which leads to the restoration of the original vesicular EEA1 population in the end of “EEA1-Cycle” and the formation of EGF/EGFR-containing vesicles that further can enter late stages of degradative pathway.

Indeed, triple-color staining of HeLa cell stimulated to internalize EGF/EGFR shows that up to 30 min of endocytosis EGFR colocalized with EEA1 in manner described earlier in this paper, but at 60 min significant portion of EGFR colocalized with lysosomal marker Lamp1. Importantly, the colocalization of EEA1 and Lamp1 was not detected (S3 Fig), that corresponds to our previous studies.

## Discussion

The vesicular appearance of membrane structures involved in endocytosis causes difficulties in determining both the principles of organization of the endocytic pathway as a whole and the boundaries between successive stages in particular. Traditionally, all vesicles containing internalized cargo are referred to as endosomes, conventionally divided into early and late ones. This should be true from the point of view of the maturation hypothesis: the cargo passes along the entire endocytic pathway in the same membrane structure that recruits “early” regulatory proteins from the cytoplasmic pool and gradually replaces them with regulators of later stages also taken from the cytoplasm. Accordingly, the regulatory proteins should always colocalize with the cargo.

However, in terms of ECV-hypothesis of stable endosomal compartments the cargo upon packing into transport vesicle called “endocytic vesicle” fuses with the pre-existing membrane structures that are the “true” early endosome/endosomes in this case, and upon processing (sorting to degradative pathway) is packed in another transport vesicles (usually called ECVs) for delivery to late endosomes. Thus, transport vesicles with cargo should be included in the early endosomal compartment and then excluded from it, without disturbing its identity and affecting the association of its membrane with the resident proteins.

Obviously, from a practical point of view, the described differences between the hypotheses of maturation and ECV should lead to the following: (i) when stimulating a synchronous wave of endocytosis, the regulatory proteins characteristic of the early stage are recruited from the cytoplasmic pool onto the membrane and at the end of this stage dissociate back in the first case, whereas in the second case the level of such membrane-associated proteins should not change; (ii) the main source of early endosomes should be the process of endocytosis, whereas in the second case it is the biosynthetic pathway, involved in biogenesis of the most of intracellular compartments; (iii) stable endosomal compartment must participate in multiple cycles of cargo transition through it.

In this paper, taken into consideration abovementioned differences, we have focused on the behavior of vesicles bearing EEA1 and have demonstrated that (i) these vesicles are detected in HeLa and A549 cells in absence of external growth factors; (ii) the level of membrane-associated EEA1 did not change after stimulation of endocytosis with EGF and formation of comparable number of EGF/EGFR-loaded vesicles; (iii) newly formed endocytic vesicles loaded with EGF/EGFR complexes directly fuse with pre-existing EEA1-positive vesicles forming hybrid structures with domain organization of united membrane, (iv) fusions results in effective increase of EGFR- positive domains area, that makes it possible to form ILVs and sort there EGF/EGFR. Later on, (v) the part of initially EGFR-positive membrane is released from the hybrid organelle in the form of EGF/EGFR-positive vesicle, that might be a transport vesicle, bearing EGF/EGFR inside in ILVs (or ECV, in terms of Gruenberg et al. [6]) to late endosomes/lysosomes. Finally, (vi) severing of Golgi but not of endocytic pathway functioning has significant effect on the number and appearance of EEA1 vesicles. BFA effect supports significant importance of TGN-derived membrane flow and exchange between endocytic biosynthetic compartments. Inhibition of trans-Golgi traffic can have many consequences associated with any endocytic pathway and affect many compartments, from the plasma membrane to endosomes and lysosomes. Therefore, we chose a 6-hour (i.e., short) period of exposure so that a certain effect could manifest itself, but would not be critical for cell life. Our main goal was to see at least some statistically significant response to the suppression of endocytic or the biosynthetic pathways, and such an answer was obtained under BFA treatment. Still, we could not say yet what happens to EEA1 protein when the membrane surface decreased. This problem requires further investigation.

The data obtained provide direct evidence of pre-existing, stable early endosomal compartment and reinforce the idea of the «classical» organization of endocytosis pathway. The population of vesicles carrying EEA1 should be considered as such early endosomal compartment. As EEA1 localizes to endosomal membranes in Rab5-dependent manner, it is widely used as the marker of early endosomal stage, identical to Rab5. However, it should be noted, that the number of Rab5-positive structures per cell is higher than those positive for the both Rab5 and EEA1 [20]. Thus, EEA1 marks a more highly specialized population of vesicles. So not all Rab5-positive vesicles are early endosomes, while Rab5^+^/EEA1^+^ might be the real early endosomes of degradative pathway.

It is generally accepted now that the main function of early endosome/endosomes is to be the first sorting step of incoming cargo for recycling back to PM, or to be delivered to trans-Golgi or to lysosomes for degradation [35,36]. Indeed, in early reports it was shown by means of EM that transferrin or BSA and EGF/EGFR complexes can be found in the same early tubulovesicular structures, but with time the latter become redistributed from the outer endosomal membrane to ILVs thus decreasing the possibility of EGFR recycling [35,37]. However, the sorting of transferrin receptors and EGFR can occur already at internalization step by recruitment them into largely different types of clathrin-coated pits that was recently demonstrated [38–40]. As a result, transferrin colocalized with the EEA1 tether only transiently at very low level and recycled back through another than EGFR endosomal structures while EGFR showed high colocalization with EEA1 and finally was delivered to Lamp1-positive lysosomes. Thus, it can be argued that the EEA1–positive endosomes is an early endosomal subcompartment specific just for the degradative lysosomal pathway. Indeed, it is possible that there are other early endosomal subcompartments regulating specific endocytic pathways like APPL/CORVET- or endotubin-positive EE [15,41,42].

Taken this into consideration we speculate that EEA1-positive early endosomes must perform function another that only sorting of incoming cargo. It is generally accepted that cargoes have to be included into ILVs of an endosome to be efficiently degraded, and this process is often identified as maturation. Obviously, the significant and fast increase in the size of endosomal membrane is necessary to form multiple intraluminal vesicles with the diameter similar to that of primary endocytic vesicles [43] and EEA1-positive structures can serve as mediators of this increase. Indeed, our live cell imaging experiments have demonstrated that EEA1-positive vesicles able to fuse with each other in absence of stimulated endocytosis that is indicative of bearing all necessary fusogenic machinery (Fig 5A, S1 Movie). Importantly, we show that newly formed EGF/EGFR-loaded vesicles directly fused with pre-existing EEA1-positive vesicles, and no fusions of only EGF/EGFR vesicles with each other were detected at least at the resolution of confocal microscope (Fig 6A and 6B, S3 and S4 Movies). Thus, the formation of such hybrid structures occurs as a result of the first heterotypic fusion that might be governed by active Rab5 both on clathrin-coated vesicle and on EE membrane [27]. Further they may accept both heterotypically fusing endocytic and EEA1-vesicles, and homotypically fusing additional EEA1 vesicles (that are real EE), as well as the hybrid EGFR/EEA1 structures. It can be concluded that EEA1 vesicles work as a platform providing the necessary machinery for rapid achievement of an excess of membrane coming from the cell surface that can be used to form ILVs of MVBs. The use of PAE A11 cells that overexpress EGFR fused to GFP, allows to clearly show that incoming membrane of endocytic vesicle does not mixed with EEA1-positive membrane being segregated into specific areas during its stay in early endosomal compartment, however EGF/EGFR-positive domains can incorporate membranes of several endocytic vesicles thus increasing the size of the domain. This process seems to be critical for EGFR-positive membrane ability to change dramatically its curvature and form ILVs. It must be noted that maintaining of domain organization of endosomes is typical for the cells with normal dynamics of MVB formation, that is visualized by light microscopy as non-pixel-to-pixel colocalization of EEA1 and cargo onto endosomes in the cells like HeLa or A549 (Fig 4, S2 Fig) and was reported earlier in many other works [6,21,44]. In addition, in the recent research M.Zerial’s group using single molecule localization microscopy combined with electron tomography (superCLEM) showed that EGF and Rab5 localized at the distinct domains of the same endosome [45].

Importantly, such mosaic domain organization allows several different process to go independently, for example, tethering/fusions, signaling, and ILVs formation. On fixed cells we demonstrated that EE vesicles contact each other with EEA1 domains, however live cell imaging of PAE A11 cells shows that it is not so easy for two endosomes to fuse: their behavior resembles a kind of dance, when two vesicles come close but fail to fuse, then go apart, than return back and finally fuse with one of them throwing towards the other a membranous protrusion, free of active EGF/EGFR complexes. Interestingly that in PAE cells EGFR-GFP localizes practically in all endolysosomal membranes, however only those receptors complexed to EGF (labeled with QD655) able to form uniform domains onto endosomal membrane. The requirement for correct domain orientation may explain relatively long period, up to 20–30 min, when infrequent multiple fusions take place during endocytosis.

Though the light microscopy does not allow ILVs identification directly, we have several reasons to argue that ILVs formation starts when EGF/EGFR are still the components of hybrid EEA1-positive endosomes. First, we demonstrated here increase in mean integral density of EGF/EGFR units, indicative of cargo concentration, is more effective during first 5–15 min after endocytosis stimulation. Second, we have shown earlier that Hrs, the first component of ESCRT0 complex initiating ILVs formation, was recruited to the EGFR-containing domain of endosomes in 5–10 min upon internalization in HeLa cells [46]. This is the agreement with EM studies reported recently [47] and supports very early beginning of MVB formation.

It should be noted, that the term “MVB” is often perceived too unambiguously, although it is obvious that whatever the point of view on the organization of the endocytotic pathway MVB is a dynamic structure that can gradually change. These alterations are usually associated with the term "maturation" and include such parameters as the number of ILVs and composition of outer membrane. It can be assumed that not all entire membrane of the initial vesicle transforms into ILV, the receptors are concentrated by ESCRT complexes in smaller areas, so that the residues of several initial vesicles united in large domain can be sufficient to form the outer MVB membrane. Also, the flow of vesicles from TGN cannot be excluded a source of replenishment of the domain area. It is widely accepted that the sorting of receptors in the early endosomes consists precisely in removing the receptor from the outer membrane into the ILVs, and this process starts in hybrid EEA1-positive endosomes. This means also that the MVB itself cannot be identified as late endosomes. In fact, as long as there are EEA1-domains in the common vesicular membrane, the vesicle remains an early endosome with a multi-vesicular appearance. Thus, our approach clearly defines the boundary of the early endosome. Actually, its function is to create effective conditions to get together the surface area of several EGF/EGFR-bearing vesicles to extent enough (i) to form numerous ILVs and (ii) cover them upon segregation with the membrane at least partially originated from PM. Importantly, we suppose that during segregation process not the portions of EEA1-positive domains are excised from hybrid structure to restore empty vesicles, but EGF/EGFR-domains collect all ILVs in its close proximity to restore then outer membrane of MVB(or ECV) filled with intraluminal vesicles. We speculate, that this active process must be mediated by some filamentous compounds like CD317/tetherin, protein that due to its structure is able to maintain mosaic membrane organization by binding edges of rafts to surrounding membrane [48] and keeping exosomes (that is a type of ILVs) in clusters upon MVE fusion with plasma membrane [43].

In many light microscopy studies endosomes of irregular shape with the size more than 700 nm are registered in the cells after 15–20 min of endocytosis is usually considering to be single membranous structures, however our study indicates that such structures most probably are clusters of smaller vesicles that able to tether by means of EEA1, but already fail to fuse. It may indicate the existence of some physical limitations that prevent formation of enormously enlarged vesicles. Indeed, recently it was reported that Rab5-GTP induces allosteric conformational change of long dimeric coil-coiled EEA1 making it flexible and then collapsed thus allowing to bring fusing membranes closer. This mechanism allows vesicles to grow, however conformation-defective EEA1 mutant produce just tethered but not fused clusters of endosomes [44]. Most probably, this process can be stopped by GTP hydrolysis on Rab5. On the other hand, it was shown that EEA1 on endosome is associated with p97, NSF-like ATPase, which inhibition also resulted in enlargement of EEA1-positive endosomes, but when active, p97 dissociate EEA1 oligomers thus preventing fusions, and decreasing tethering [49]. Authors propose that p97 ATPase may be involved in regulation the size of early endosomes by governing the oligomeric state of EEA1, however it needs further work to get together all elements of the mosaic.

Described here two- and multidomain EEA1-positive structures, as well as their clusters were detected by many authors in diverse types of cells, but they did not identify them as meaningful. This work is the first, to our knowledge, that describes the strict order of these consecutive steps typical for EE functioning.

Summarizing, we provide direct evidence for stable, recovering early endosomal compartment that play, except sorting, important role in processing cargoes destined for lysosomal degradation. Physically, it means removing cargo molecules from outer membrane of vesicle to avoid recycling and introducing them in intraluminal vesicles that must be form from the same outer membrane. Pre-existing EEA1-endosomes, bearing all necessary fusing machinery, efficiently allow creating excess of membrane and getting together membranes of several incoming vesicles thus providing possibility for cargo-containing domain to form ILVs with help of ESCRT-complexes. Thus, at enter sites EEA1-endosome meets small cargo-loaded vesicle and at exit site produce MVB (or premature MVB) with cargo inside ready to meet lysosomes possibly after series of homotypic MVB-MVB fusions or some other sorting events. However, EEA1-vesicular population behavior exactly matches the definition of a stable compartment that maintains its identity while passing cargo flows through it. Interestingly, the same way of action was proposed for lysosomes by Paul Luzio, with formation and segregation of hybrid endolysosomes [50,51].Taking all this into account we can suggest that the main principles of vesicular traffic organization is the same for every transport pathway, the only specificity of endocytic flow is that its compartments have vesicular appearance.

## Materials and methods

### Cell culture

HeLa and A549 cells were obtained from the Russian Cell Culture Collection (Institute of Cytology RAS, Russia). Porcine aortic endothelial cells PAE clone A11, stably transfected with plasmid encoding EGFR fused with GFP at C-terminus, were a generous gift from A.D. Sorkin (University of Pittsburgh, USA). The cells were maintained in DMEM supplemented with 10% fetal bovine serum (PAA, Austria) at 37°C in the atmosphere of 5% CO2. The medium for PAE A11 cells was additionally supplemented with 500 μg/ml genecitin (G418, Sigma-Aldrich, USA) as selection agent. The cells were grown up to approximately 70% of monolayer and then serum-starved in DMEM containing 0.1% serum for 12 h before experiment.

### Cell transfection

For live-cell imaging HeLa cells were transiently transfected with plasmid encoding N-terminal GFP-tagged EEA1 protein (Addgene plasmid 42307, USA) using Lipofectamine 2000 (Invitrogen, USA) according to the manufacturer protocol. Briefly, the cells were split on chambered coverglasses with the bottom area 2 cm^2^ (Nunclon Surface, Denmark) to be 70% confluence at the day of transfection. Plasmid DNA (0.5 μg) in 25 μl of DMEM was mixed with 25 μl DMEM medium, containing 1.2 μl Lipofectamine 2000 (Invitrogen, USA). A mixture was incubated at room temperature for 10 min and then added to cells, cultivated in 150 μl of fresh growth medium to a total volume of 200 μl. The cells were incubated in such medium for 24 hours, after that medium was changed to growth medium containing 0.1% serum for 12 h before experiment. Transfection does not affect in any way the normal behavior of endosomes as the results obtained on fixed EEA1-GFP expressing cell was the same as on fixed cells without the plasmid.

### Stimulation of endocytosis

The cells were grown on 10x10 mm coverslips for immunofluorescence staining experiments, in chambered coverglasses (NunclonSurface, Denmark) for live imaging experiments or on 100 mm plastic dishes for cell lysate preparation. The cells were serum-starved for 12 h before experiment and then endocytosis was stimulated according to two different protocols:

#### Stimulation of endocytosis by pulse-chase

The cells were rinsed with warm (37°C) DMEM supplemented with 20 mM HEPES and 1% BSA (working medium, WM). For immunofluorescence and cell lysate preparation endocytosis was stimulated with 40 ng/ml recombinant EGF (Sigma-Aldrich, USA) in fresh WM at 37°C, added to the cells for 5 min. For the live imaging experiments 4 nM of biotinylated EGF (Invitrogen, USA) was conjugated with 30-fold excess of streptavidine-Cy3 (Invitrogen, USA) or 1 nM streptavidine-quantum dots with emission maximum at 655 nm (QD655, Invitrogen, USA) by 30 min incubation with constant shaking at 37°C. Then the mixture was added to the cells for 5 min. After that, in both cases, unbound ligand was washed out for several times with warm WM and the incubation was continued up to the time indicated at 37°C.

#### Stimulation of endocytosis according to EGF pre-binding protocol

The cells were washed twice with cold (4°C) WM and placed on ice. Ice-cold WM, containing EGF or biotinylated EGF (in case of live imaging experiments) were added to the cells for 40 min in concentrations mentioned above. Unbound ligand was then washed out for several times with cold WM. In the case of non-biotinylated EGF endocytosis was stimulated by shifting the cells to 37°C for the time indicated. For the live imaging experiments streptavidine-Cy3 was added to the cells in concentration mentioned above for 20 min, 4°C. After this, unbound fluorescence label was washed out for several times with cold WM and endocytosis of biotinylated EGF was stimulated by shifting the cells to 37°C for the time indicated.

### Inhibitors

Nocodazole (Sigma-Aldrich, USA) treatment was carried out during 30 min using the concentration of 20μM, to disrupt the microtubules. For the inhibition of the endocytic pathway the cells were treated with the inhibitor of dynamin-dependent endocytosis dynasore (80 μM, Sigma-Aldrich, USA) alone or in combination with fluid-phase endocytosis inhibitor 5-(N, N-hexamethylene) amiloride (66 μM, Sigma-Aldrich, USA). Brefeldin A (ICN Biomedicals, USA) was used in concentration 10 mg/ml as the inhibitor of the biosynthetic pathway. The cells were incubated in DMEM containing indicated inhibitors for 6 h.

### Immunofluorescence

The cells were grown on coverslips for achieving about 70% monolayer at the day of experiment. Endocytosis was stimulated as described above. At the time indicated after stimulation the cells were fixed with 4% formalin for 15 min at the room temperature (RT). After that the cells were washed with PBS and then permeabilized with 0.5% Triton X-100 for 15 min at RT. For Lamp1 labelling cells were permeabilized with 0.05% Brij56 (Sigma-Aldrich, USA) and all the washing steps was conducted using detergent-free PBS. After washing the cells were blocked in 1% BSA for 30 min at RT to prevent non-specific antibody binding. Then the cells were incubated overnight at 4°C with the following primary antibodies, diluted in 1% BSA: polyclonal rabbit antibodies against the intracellular domain of EGFR (Cell Signaling, USA) or against C-terminal end of EEA1 (Abcam, UK) were used at a dilution 1:100 or 1:1000, respectively; monoclonal mouse antibodies against N-terminal end of EEA1 (Transduction Lab, USA) or Lamp1 (Abcam, UK) were used at a dilution 1:200 or 1:50, respectively. After washing with PBS containing 0.1% Tween-20 (BioRad, USA) the cells were incubated with 5 μg/ml Hoechst 33342 (Molecular Probes, USA), secondary antibodies GAR-Alexa Fluor 568 (Molecular Probes, USA) and GAM-Alexa Fluor 488 (Molecular Probes, USA) at a dilution 1:200 for 20 min at 37°C. Finally the cells were mounted in 0.2 M DABCO (1,4-diazabicyclo(2.2.2) octane) glycerol-containing media.

### Laser confocal scanning microscopy

Distribution of fluorescently labeled proteins in cells was analyzed by confocal laser scanning microscope Leica TCS SP5 (Leica Microsystems, Germany) with oil immersion objective 40X, 1.25 NA. The fluorescent signal from Hoechst 33342 and QD655 was exited using a diode 405 laser; from Alexa 488 or GFP using argon 488 laser and from Alexa 568 or Cy3 using He-Ne 543 laser. Whole-cell z-scanning was conducted with 500 nm step for subsequent quantitative analysis.

Live-cell imaging was conducted using Leica TCS SP5 and thermostatic chamber, using oil immersion objective 40X, 1.25 NA. The base plane of the cell, where the majority of endosomes were located, was imaged with the scanning frequency 400–700 Hz and image size of 1024x1024 pixels, unless other is indicated. Fluorescence images were acquired every 0.7–5.8 s.

### Image analysis

The number of vesicles and its parameters were evaluated using ImageJ 1.40 g (National Institute of Health, USA). 8-bit maximal intensity projections of a z-stack series were taken into analysis. Spatial calibration of images was conducted using «Set Scale» function. Segmentation was performed as described previously [52], with some modifications. Median filter with the radius value 6 for green channel and 10 for red channel (values were matched experimentally) was used and obtained image was subtracted from the initial one. As the result, the difference between the vesicle edge pixel intensity and adjoining background pixel intensity was larger at the obtained image compared with the initial one. This allowed avoiding the artificial enlargement of vesicle size upon image binarization. Then obtained image was binarized using the threshold values that provided the maximal correspondence between the original and binarized image. Regions Of Interest (ROI) were selected with the use of «Analyze Particles» function, so that ROI corresponded to vesicles, whose area were larger than 20000 nm^2^. Obtained selections were restored on the initial image and used for measuring integral density of each vesicle and the total area of all the vesicles per cell.

To evaluate the object-based colocalization the same segmentation procedure was used on the single optical slice, representing the base plane of the cell. Then the number of overlapping objects was evaluated by superposition of ROIs from one channel to the binarized image of another channel. The values presented are the percentage of the number of overlapping objects from the total number of objects.

### Isolation of cell membrane fraction

EGF was added to the cells according to pre-binding protocol and 15, 30, 60, 90 min after shifting of the cells to 37°C for initiation of endosomes formation cell membrane fraction was isolated by ultracentrifugation. Briefly, the cells were incubated in TES buffer (pH = 6.0), containing 20 mM triethanolamine hydrochloride; 0.25 M sucrose; 1mM EDTA. Cell suspension was passed several times through the syringe and underwent centrifugation for 15 min at 10 000g for removing of undestroyed cells, nucleus and mitochondria. Then supernatant was centrifuged again for 30 min at 100 000g for isolation of cell membrane fraction that was analyzed by western blotting.

### Western blotting

For western blot analysis samples were denatured by the addition of SDS-containing sample buffer and run on 7.5–12.5% polyacrylamide gels according to Laemmli [53]. Western blotting analysis was provided according to ECL Western blotting protocol (Amersham, Sweden).

We used rabbit polyclonal anti EGFR (Cell Signaling, USA) antibodies at a dilution 1:1000, rabbit polyclonal anti-glyceraldehyde-3-phosphate dehydrogenase (GAPDH) (Cell Signaling, USA) antibodies at a dilution 1:1000. Primary antibodies were diluted in TBS buffer (10 mM Tris; 74 mM NaCl, 0,01M PBS) containing 0.1% Tween (T-TBS) and 5% BSA. Mouse monoclonal anti-EEA1 (Transduction Lab, USA) antibodies were diluted in T-TBS, containing 5% non-fat milk (1:5000). The nitrocellulose membranes were incubated with primary antibodies containing medium overnight at 4°C. The blots were rinsed 5 times for 5 min with T-TBS buffer followed by their incubation for 1 h at room temperature with goat anti-rabbit or goat anti-mouse HRP-conjugated secondary antibodies (Cell Signaling, USA), diluted in T-TBS, containing 5% non-fat milk (1:10000).

The densitometry was conducted using Image Lab software (Bio-Rad Laboratories, USA). Ponceau S density was used as the loading control and then fold change of normalized EEA1 density was quantified relative to the unstimulated cells. Three biological replicates were quantified. To evaluate the statistical difference we used non-parametric one-sample sign test with Bonferroni-Holm correction for multiple comparisons.

### Statistics

For each time point 15–20 cells were taken for analysis. The cells presented have typical (>90% of population) appearance for certain conditions. Each experiment was repeated at least three times and the results of typical experiment were presented if other is not indicated. Data analysis was carried out using R software [54]. To validate the statistical significance, the non-parametric Mann-Whitney U-test for independent samples was used with Bonferroni-Holm correction for multiple comparisons if other is not indicated.

## Supporting information

S1 Fig The original images of western blots. The red frames show regions used for the indicated figure.
(TIF)

S2 Fig The dynamics of EEA1- and EGFR-positive vesicles colocalization in A549 cells following the stimulation of EGF/EGFR-complexes endocytosis. Endocytosis in A549 cells was stimulated according to pulse-chase protocol by adding of EGF for 5 min followed by washout of unbound ligand and chase period at 37°C. Cells not treated with ligand (- EGF) and cells chased for the indicated period were fixed and immunostained using antibodies against EEA1 (green channel) and EGFR (red channel). (A) Maximum intensity projections of the typical cells are presented. Scale bar—10 μm (3 μ in the enlarged insets). The number (B), mean integral density of vesicles (C) and object-based colocalization of the cells from the same experiment (D, E) were quantified. For each time point 15–20 cells were analyzed. The data are presented as boxplot that shows median, 25% and 75% quartiles, minimum and maximum value. In B,C the star indicates significant difference from unstimulated cells (p<0.05).
(TIF)

S3 Fig The localisation of EEA1 and Lamp1 during EGFR endocytosis. Endocytosis in HeLa cells was stimulated according to pulse-chase protocol by adding of EGF-Cy3 (red channel) for 5 min followed by washout of unbound ligand and chase period at 37°C. Cells were fixed and immunostained using antibodies against EEA1 (green channel) and Lamp1 (blue channel). Maximum intensity projections 3 optical slices of typical cell are presented. Arrows indicates the EEA1/EGF-containing hybrid vesicles and star indicate the cluster of such vesicles; arrowheads show Lamp1/EGF-positive vesicles. Scale bar—10 μm.
(TIF)

S1 Movie Fusion of EEA1-positive endosomes in serum-starved cells. Serum-starved HeLa cells transiently expressing EEA1-GFP were imaged at +37°C with 1.39 sec interval between frames. The time after start of imaging is indicated at the frames. Scale bar– 3 μm. Frame rate– 4 fps.
(MP4)

S2 Movie Fission of EEA1-positive endosomes in serum-starved cells. Serum-starved HeLa cells transiently expressing EEA1-GFP were imaged at +37°C with 0.7 sec interval between frames. The time after start of imaging is indicated at the frames. Scale bar– 3 μm. Frame rate– 8 fps.
(MP4)

S3 Movie Formation of hybrid EGF/EEA1-endosome. EGF-Cy3 (red) endocytosis in HeLa cells transiently expressing EEA1-GFP (green) was stimulated according to the pre-binding protocol, described in the Material and methods section. Time-lapse imaging at +37°C was started in 15 min after endocytosis stimulation with 5.8 sec interval between frames. The time after start of imaging is indicated at the frames. Scale bar– 3 μm. Frame rate– 5 fps.
(MP4)

S4 Movie Fusion of hybrid endosomes. EGF-Cy3 (red) endocytosis in HeLa cells transiently expressing EEA1-GFP (green) was stimulated according to the pre-binding protocol, described in the Material and methods section. Time-lapse imaging at +37°C was started in 15 min after endocytosis stimulation with 5.8 sec interval between frames. The time after start of imaging is indicated at the frames. Scale bar– 3 μm. Frame rate– 5 fps.
(MP4)

S5 Movie Fusion of hybrid endosomes in PAE-A11 cells. EGFR endocytosis was stimulated according to pulse-chase protocol by adding of EGF-QD655 (red) for 5 min to PAE-A11 cells expressing EGFR-GFP (green) followed by time-lapse imaging at +37°C with 4.95 sec interval between frames. The time after start of imaging is indicated at the frames. Scale bar– 3 μm. Frame rate– 5 fps. Arrows point out EGFR-positive tubule between vesicles ready to fuse.
(MP4)

S6 Movie Aggregated hybrid endosomes. EGF-Cy3 (red) endocytosis in HeLa cells transiently expressing EEA1-GFP (green) was stimulated according to the pre-binding protocol, described in the Material and methods section. Time-lapse imaging at +37°C was started in 41 min after endocytosis stimulation with 5.8 sec interval between frames. The time after start of imaging is indicated at the frames. Scale bar– 3 μm. Frame rate– 5 fps.
(MP4)

S7 Movie Aggregated hybrid endosomes in PAE-A11 cells. EGFR endocytosis was stimulated according to pulse-chase protocol by adding of EGF-QD655 (red) for 5 min to PAE-A11 cells expressing EGFR-GFP (green) followed by time-lapse imaging at +37°C started in 27 min after endocytosis stimulation with 4.92 sec interval between frames. The time after start of imaging is indicated at the frames. Scale bar– 3 μm. Frame rate– 7 fps.
(MP4)

S8 Movie EGF-vesicle segregation from hybrid endosome. EGF-Cy3 (red) endocytosis in HeLa cells transiently expressing EEA1-GFP (green) was stimulated according to the pre-binding protocol, described in the Material and methods section. Time-lapse imaging at +37°C was started in 27 min after endocytosis stimulation with 5.8 sec interval between frames. The time after start of imaging is indicated at the frames. Scale bar– 3 μm. Frame rate– 5 fps.
(MP4)
