# Supplementary figures and images for: Functional cycle of EEA1-positive early endosome: Direct evidence for pre-existing compartment of degradative pathway
Source: PLoS One. 2020 May 1;15(5):e0232532. doi: 10.1371/journal.pone.0232532 (PMC7194439; doi:10.1371/journal.pone.0232532)

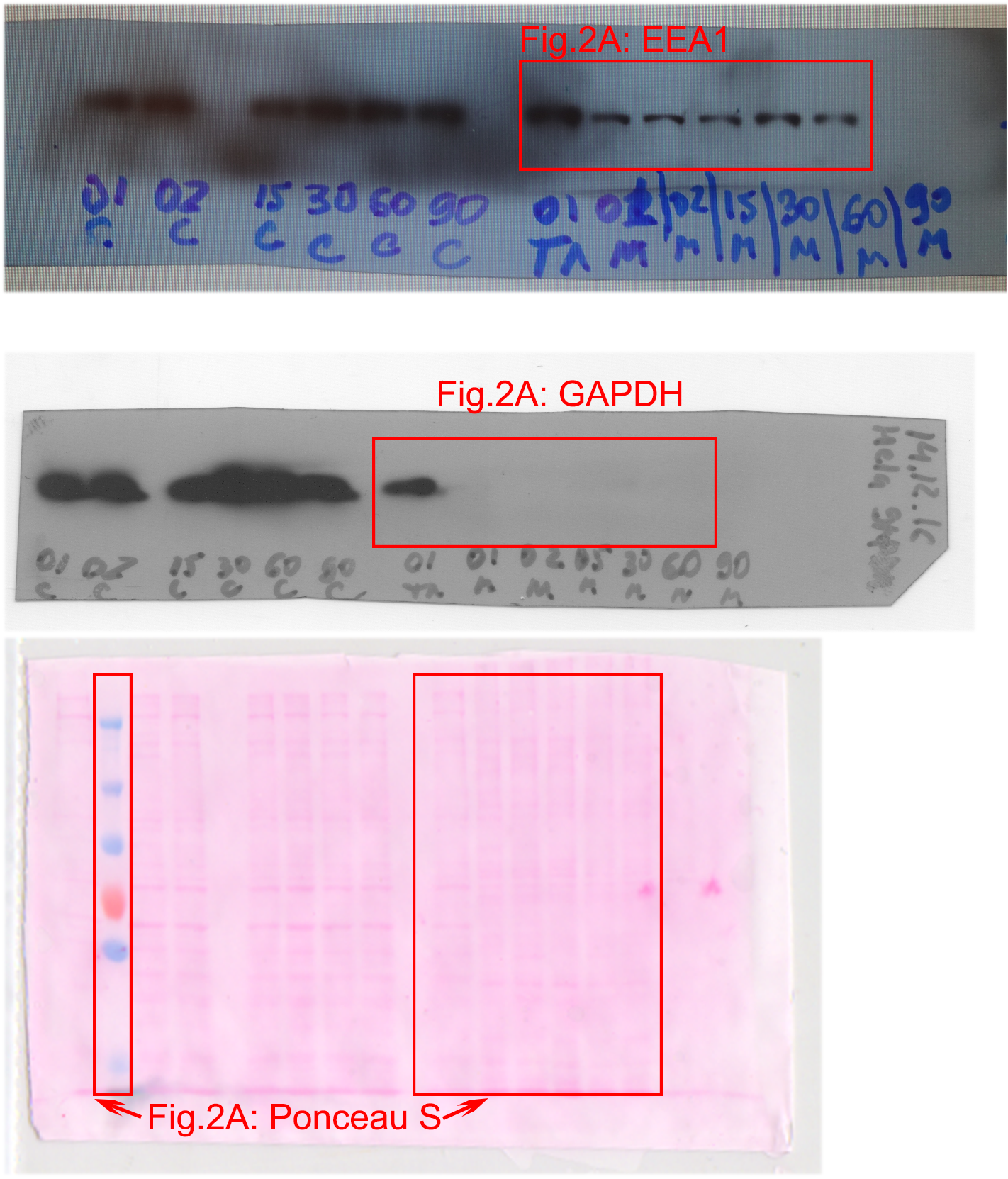

Supplement: S1 Fig — The red frames show regions used for the indicated figure. (TIF) [file pone.0232532.s001.tif]

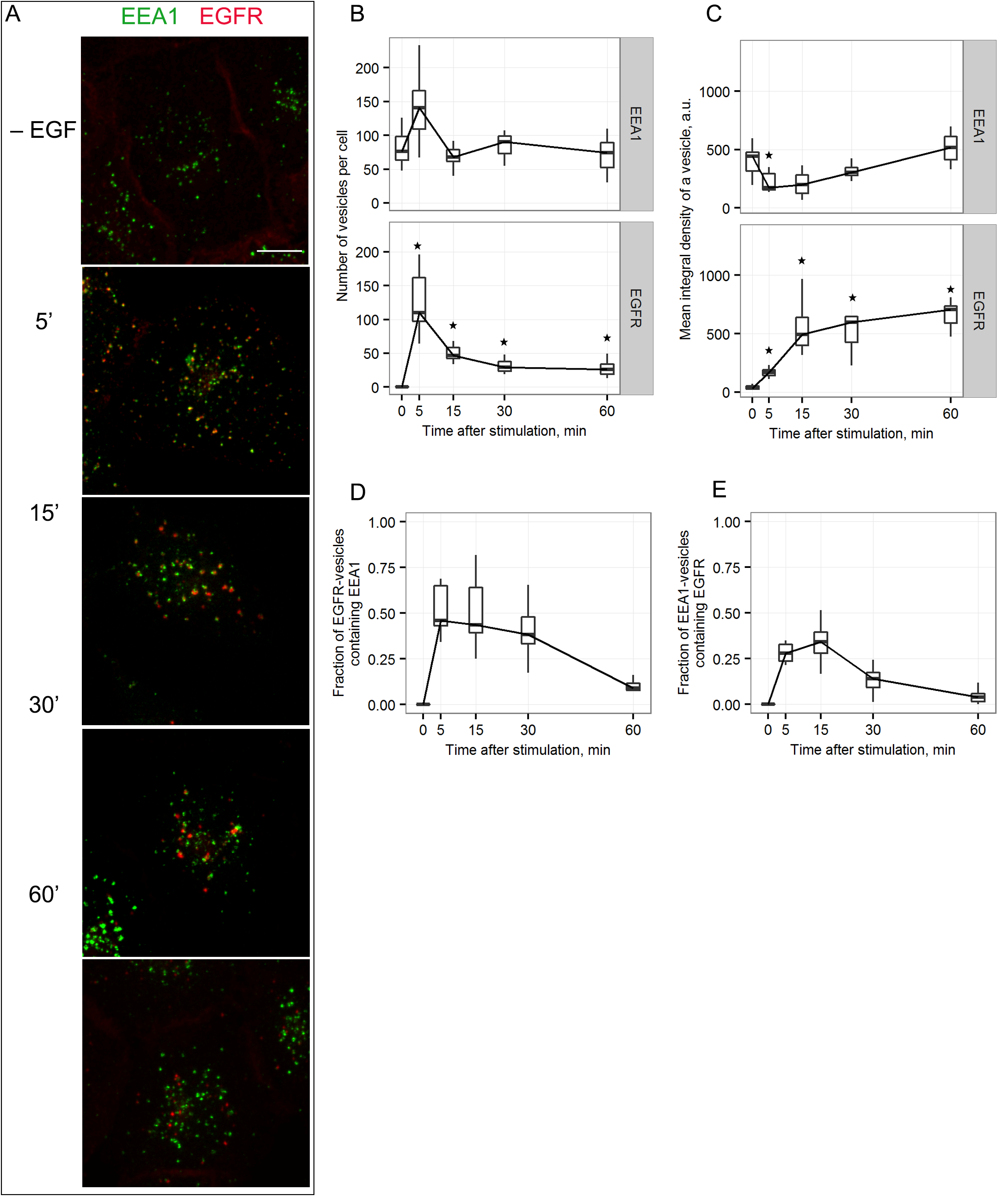

Supplement: S2 Fig — Endocytosis in A549 cells was stimulated according to pulse-chase protocol by adding of EGF for 5 min followed by washout of unbound ligand and chase period at 37°C. Cells not treated with ligand (- EGF) and cells chased for the indicated period were fixed and immunostained using antibodies against EEA1 (green channel) and EGFR (red channel). (A) Maximum intensity projections of the typical cells are presented. Scale bar—10 μm (3 μ in the enlarged insets). The number (B), mean integral density of vesicles (C) and object-based colocalization of the cells from the same experiment (D, E) were quantified. For each time point 15–20 cells were analyzed. The data are presented as boxplot that shows median, 25% and 75% quartiles, minimum and maximum value. In B,C the star indicates significant difference from unstimulated cells (p<0.05). (TIF) [file pone.0232532.s002.tif]

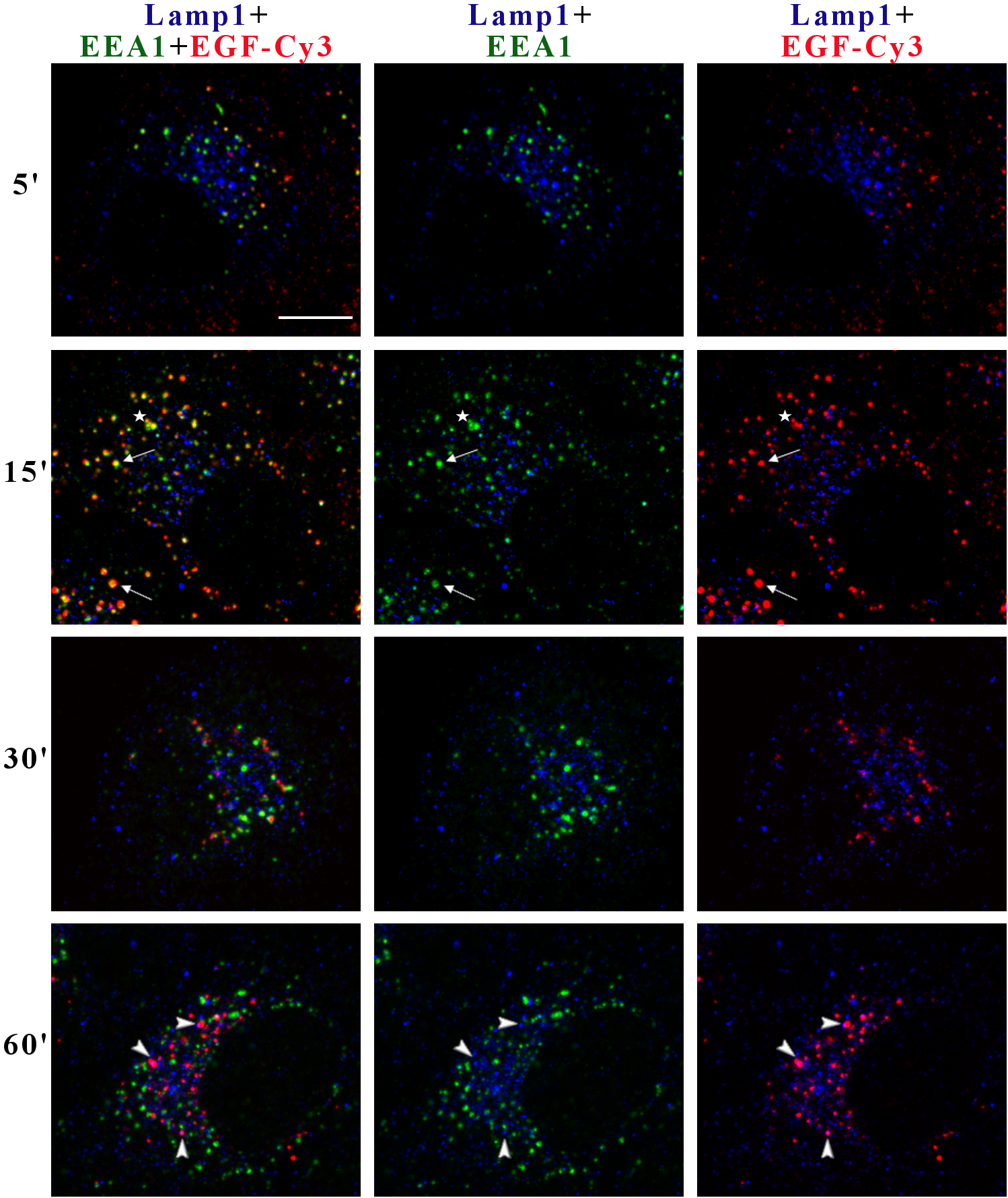

Supplement: S3 Fig — Endocytosis in HeLa cells was stimulated according to pulse-chase protocol by adding of EGF-Cy3 (red channel) for 5 min followed by washout of unbound ligand and chase period at 37°C. Cells were fixed and immunostained using antibodies against EEA1 (green channel) and Lamp1 (blue channel). Maximum intensity projections 3 optical slices of typical cell are presented. Arrows indicates the EEA1/EGF-containing hybrid vesicles and star indicate the cluster of such vesicles; arrowheads show Lamp1/EGF-positive vesicles. Scale bar—10 μm. (TIF) [file pone.0232532.s003.tif]
